# The association of diabetes mellitus treated with oral antidiabetic drugs and insulin with mortality after transcatheter valve implantation: a 3-year follow-up of the TAVIK registry

**DOI:** 10.1186/s12933-019-0873-6

**Published:** 2019-05-28

**Authors:** Panagiotis Tzamalis, Valentin Herzberger, Jens Bergmann, Alexander Wuerth, Peter Bramlage, Holger Schroefel, Claus Schmitt, Gerhard Schymik

**Affiliations:** 1 Medical Clinic IV-Department of Cardiology, Municipal Hospital Karlsruhe, Academic Teaching Hospital of the University of Freiburg, Moltkestr. 90, 76133 Karlsruhe, Germany; 2 Medical Clinic III-Department of Cardiology, Vincentius Hospital Karlsruhe, Karlsruhe, Germany; 3 Institute for Pharmacology and Preventive Medicine, Cloppenburg, Germany; 4 Department Cardiovascular Surgery, University Heart Center Freiburg-Bad Krozingen, Bad Krozingen, Germany

**Keywords:** Valvular heart disease, TAVI, Diabetes mellitus

## Abstract

**Background:**

Diabetes mellitus (DM) on insulin is a patient-related factor in the assessment of surgical risk based on the EuroSCORE II and, as such, it confers additional risk on outcomes after transcatheter aortic valve implantation (TAVI). The aim of this study was to investigate the effect of diabetes mellitus treated with insulin and oral antidiabetic drugs on clinical outcomes after TAVI.

**Methods:**

This study is an analysis of 2000 patients who underwent TAVI between 2008 and 2015. Patients were stratified post hoc into the following categories: without diabetes (n = 1337), with diabetes treated with oral antidiabetic drugs (OAD; n = 387) and with diabetes treated using insulin (n = 276).

**Results:**

There was no significant difference in device success (89.5% vs 89.4% vs 88.8%, adjusted odds ratio (adjOR) 1.10 [95% confidence interval (CI) 0.64–1.91]) and VARC-2-defined major complications among the three groups of patients (without DM, OAD, and insulin, respectively). Minor but not major or disabling strokes (adjOR 2.19; 95% CI 1.11–4.3) and overall renal complications (but not stage 2/3 alone) (adjOR 1.46; 95% CI 1.18–1.81) were more common in patients with diabetes than in those without diabetes. Insulin-treated patients had a significantly lower survival rate than that of patients with orally treated diabetes and of those without diabetes at 1 year (75.7% vs. 84.5% vs 84.7%, pairwise p < 0.01) and 3 years (56.9% vs. 65.9% vs. 67.9%, adj. p < 0.05) after TAVI. However, insulin-treated diabetes was not identified as an independent risk factor for higher mortality in the first (HR 1.29; 95% CI 0.97–1.72, p = 0.084) and 3rd years (HR 1.21; 95% CI 0.98–1.49; p = 0.079) after multivariable adjustment.

**Conclusions:**

Although insulin-dependent DM is an established component of surgical risk assessment, it was not identified as an independent factor associated with reduced survival in TAVI. DM treated with oral antidiabetic drugs or insulin may have less role in decision making of treatment in TAVI candidates.

## Background

Transcatheter aortic valve implantation (TAVI) has been established as a standard of care for patients with severe aortic stenosis [1–4] with good clinical outcomes compared to those of surgical aortic valve replacement [5–8]. A deeper understanding of the role and pathophysiology of the comorbidities of these patients has improved the risk assessment and management of complications after TAVI [9–11]. Diabetes mellitus (DM) is a systemic disease and a risk factor for cardiovascular events [12]. Studies have additionally suggested a role for the systemic inflammation of diabetes in the progression of aortic stenosis [13, 14]. However, the implications of DM on the procedural outcomes of TAVI remain controversial. In particular, some studies suggest a minor effect of diabetes on this procedure [15] while others significantly worse 30-day [16] and 1-year outcome [17–19]. This contradiction is also reflected in the current risk assessment scores. More specifically, the logistic EuroSCORE I does not include the patient factor diabetes, whereas the Society of Thoracic Surgery (STS) Score utilizes the patient factor diabetes, independently of the applied therapy; in the EuroSCORE II, DM on insulin is a patient-related factor considered to have satisfactory performance in predicting perioperative as well as 30-day mortality [20–22]. The aim of this study was to investigate the effect of diabetes mellitus treated with insulin and oral antidiabetic drugs on clinical outcomes after TAVI.

## Methods

The present report is a subanalysis of the prospective, single-center TAVIK (TAVI team Karlsruhe) registry, the methodology of which has been previously described [23, 24]. The registry contains data on 2000 patients with severe aortic stenosis who underwent TAVI in Karlsruhe between April 2008 and March 2015, with a subsequent follow-up of 3 years. The responsible local ethics committee (Stuttgart, Germany) approved this study, and written informed consent was obtained from each patient prior to their inclusion.

### Procedures and devices

Prior to the intervention, cardiac catheterization, angiographic cardiac and peripheral vessel computed tomography, and transesophageal echocardiography were performed. The results were carefully assessed for factors that may affect the intervention [23]. TAVI was performed by a fully trained multidisciplinary team (an interventional cardiologist, a cardiac surgeon, and an anesthesiologist specializing in cardiac surgery) with support from the catheterization lab and operating room staff proficient in transcatheter procedures. The choices of access route, prosthesis and anesthesia type were made on a patient-by-patient basis.

### Data collection and variables

Data regarding patient demographics (age, gender), disease characteristics and surgical risk factors prior to the intervention were collected. Clinical characteristics included information on overall comorbidities of the patients such as chronic lung disease, renal impairment, extracardiac arteriopathy, previous cardiac surgery, endocarditis, recent myocardial infarction, mitral valve disease and pulmonary hypertension. Further information concerned the NYHA stage of the patients as well as the urgency of the procedure. Finally, periprocedural details and outcomes, 30-day safety outcomes and mortality over 1 year were collected prospectively for TAVI patients. Follow-up was conducted during outpatient visits or via structured telephone interviews.

### Endpoints

The primary objective was to compare the survival rates of patients without diabetes to those of patients with diabetes (orally or insulin treated) at 30 days, 1 year and 3 years after the intervention. The key secondary outcome was the proportion of patients meeting the Valve Academic Research Consortium (VARC)-2 [25] early safety endpoint at 30 days (composite of all-cause mortality; all stroke; life-threatening bleeding; acute kidney injury [AKI] stage 2/3, including renal replacement therapy; coronary artery obstruction requiring intervention; major vascular complication and/or valve-related dysfunction requiring a repeat procedure (balloon aortic valvuloplasty, TAVI or surgical aortic valve replacement [SAVR]). Other outcomes included composite elements considered individually and VARC-2-defined device success, including the absence of procedural mortality, correct positioning of a single prosthetic heart valve into the proper anatomical location, intended performance of the prosthetic valve (aortic valve gradient < 20 mmHg) and no moderate or severe prosthetic valve regurgitation.

### Statistical analysis

Continuous variables are presented as the mean ± standard deviation (SD) and were compared using a t-test. Categorical variables are presented as absolute numbers with frequencies (%) and were compared using a χ^2^ test (or Fisher’s exact test where appropriate). The odds of periprocedural complications and 30-day safety events are presented as odds ratios (OR) with 95% confidence intervals (CI) and p-values. The inverse probability of treatment weighting was utilized to account for baseline imbalances between the two groups, with adjustments for age, ejection fraction, previous coronary artery bypass grafting (CABG), coronary artery disease, major neurological deficits, renal disease and frailty between patients without and with DM. The adjustment was performed by building a model using a regression analysis with these factors and the predicted probability of treatment weighting was applied in subsequent analyses. No adjustment was performed in the event of zero cases. Survival was evaluated using Kaplan–Meier curves and life tables, with the survival distributions of the two samples compared using a log-rank test. Stepwise Cox regression analysis was performed with the enter method to identify risk factors of mortality in the overall TAVI population. A covariate was removed from the model if the p-value exceeded 0.10.

All tests were two-sided, and a p-value of < 0.05 was considered statistically significant. Data analyses were conducted using SPSS version 20.0 (IBM, Chicago, IL, USA).

## Results

### Patient characteristics

The patient characteristics are presented in Table 1. Of the 2000 analyzed patients, 387 (19.3%) had orally treated DM, and 276 (13.8%) had insulin-treated DM. Patients with DM were younger than those without DM (82.3 years for patients without DM vs 81.4 years for patients with orally treated DM vs 79.8 ± 5.5 years for patients with insulin-treated DM, p < 0.001) and had a lower ejection fraction (57.6 vs 56.4 vs 53.9, p = 0.001 respectively) and a higher incidence of comorbidities, such as coronary artery disease (57.4% vs 64.9% vs 72.1%, p < 0.001), previous CABG (12.9% vs 16.0% vs 24.6%, p < 0.001), major neurological deficits (9.6% vs 8.3% vs 14.5%, p = 0.021) and renal failure (4.0% vs 6.5% vs 10.9%, p < 0.001). Patients with diabetes had a higher degree of frailty (30.8% vs 36.4% vs 41.3%, p < 0.001) as well as a higher EuroSCORE I (20.5 vs 20.6 vs 24.7, p = 0.015) than patients without DM.

**Table 1:** Patient characteristics

	Without diabetes (n = 1337)	Diabetes on OAD (n = 387)	Diabetes on insulin (n = 276)	p-value
Age (years)	82.30 ± 5.51	81.35 ± 5.12	79.75 ± 5.49	< 0.001
Male gender	590 (44.1)	182 (47.0)	140(50.7)	0.110
LVEF (%)	57.57 ± 13.07	56.37 ± 13.77	53.85 ± 14.63	0.001
PAD	187 (14.0)	60 (15.5)	71 (25.7)	0.067
CAD	767 (57.4)	251 (64.9)	199 (72.1)	< 0.001
Previous CABG	173 (12.9)	62 (16.0)	68 (24.6)	< 0.001
Previous MI	137 (10.2)	49 (12.7)	39 (14.1)	0.110
COPD (moderate/severe)	150 (11.2)	52 (13.4)	33 (12.0)	0.488
Mitral valve disease (> II°)	174 (13.0)	48 (12.4)	38 (13.8)	0.875
Porcelain aorta	88 (6.6)	26 (6.7)	14 (5.1)	0.621
Log. EuroSCORE I	20.46 ± 14.52	20.60 ± 15.12	24.69 ± 18.17	< 0.001
Carotid stenosis	231 (17.3)	74 (19.1)	64 (23.2)	0.065
Previous valve surgery	46 (3.4)	14 (3.6)	5 (1.8)	0.344
Major neurological deficits	128 (9.6)	32 (8.3)	40 (14.5)	0.021
Critical perioperative situation	19 (1.4)	3 (0.8)	6 (2.2)	0.310
Pulmonary hypertension (moderate/severe)	277 (20.7)	68 (17.6)	69 (25.0)	1.0
Renal failure incl. dialysis	53 (4.0)	25 (6.5)	30 (10.9)	< 0.001
Emergency case	15 (1.1)	7 (1.8)	6 (2.2)	0.157
Overall frailty	412 (30.8)	141 (36.4)	114 (41.3)	0.001
NYHA IV	88 (6.6)	27 (7.0)	32 (11.6)	0.014

*CABG* coronary artery bypass graft, *CAD* coronary artery disease, *LVEF* left ventricular ejection fraction, *MI* myocardial infarction, *PAD* peripheral artery disease, *COPD* chronic obstructive pulmonary disease

### Procedural characteristics

A SAPIEN valve was implanted in 17.7% of patients, while SAPIEN XT was implanted in 42.8%, SAPIEN 3 was implanted in 16.4%, CoreValve was implanted in 18.0%, Symetis was implanted in 4.3%, and JenaValve/Portico was implanted in 1.0%. There was no statistically significant difference in the procedural characteristics between patients without DM and those with orally treated and insulin-treated DM concerning the use of balloon or self-expandable valves (77.8% vs 79.7% vs 72.1%, p = 0.126 for the balloon expandable valves) and access route (64.8% vs 64.9% vs 63.0%, p = 0.844 for the transfemoral access).

### Periprocedural outcomes and early safety

VARC-2-defined device success [25] was achieved in 89.5% of patients without diabetes, 89.4% of patients with orally treated diabetes and 88.8% of patients with insulin-treated diabetes (p = 0.944). Overall, there were low rates of procedural mortality (1.3% vs 1.3% vs 2.5%, p = 0.685).

30-day mortality rate was comparable between patients with and without DM (5.1% vs 4.4% vs 7.6%, adjusted p = 0.814 for total mortality, 3.4% vs 3.9% vs 5.4%, adjusted p = 0.711 for cardiovascular mortality). Statistically significant complication rates were identified for minor strokes (3.7% vs 1.6% vs 1.8%, adjusted p = 0.023) as well as for renal complications (22.6% vs 29.7% vs 36.6%, adjusted p < 0.001) but not for stage 2 and 3 renal failure (2.7% vs 3.4% vs 4.7%, adjusted p = 0.248, Table 2). The VARC-2 safety endpoint was met by 87.1% vs 84.2 vs 84.8%, adjusted p = 0.096 (Table 3).

**Table 2:** VARC-2 complications (30 days)

	Without diabetes (n = 1337)	Diabetes on OAD (n = 387)	Diabetes on insulin (n = 276)	p-value	HR (95% CI)*	p-value*
Total mortality	68 (5.1)	17 (4.4)	21 (7.6)	0.255	1.05 (0.69–1.60)	0.814
Cardiovascular death	45 (3.4)	15 (3.9)	15 (5.4)	0.212	0.91 (0.56–1.48)	0.711
Noncardiovascular death	23 (1.7)	2 (0.5)	6 (2.2)	0.160	1.46 (0.64–3.36)	0.369
Myocardial infarction	14 (1.0)	7 (1.8)	4 (1.4)	0.379	0.58 (0.26–1.33)	0.201
Stroke	78 (5.8)	14 (3.6)	13 (4.7)	0.110	1.49 (0.94–2.35)	0.088
Major/disabling stroke	29 (2.2)	8 (2.1)	8 (2.9)	0.731	0.94 (0.50–1.76)	0.854
Minor stroke	49 (3.7)	6 (1.6)	5 (1.8)	0.046	2.19 (1.11–4.3)	0.023
Bleeding	254 (19.0)	71 (18.3)	58 (21.0)	0.809	0.96 (0.75–1.22)	0.735
Life-threatening or major	127 (9.5)	33 (8.5)	31 (11.2)	0.936	1.02 (0.73–1.46)	0.927
Minor	127 (9.5)	38 (9.8)	27 (9.8)	0.872	0.91 (0.66–1.26)	0.584
Renal complications	302 (22.6)	115 (29.7)	101 (36.6)	< 0.001	1.46 (1.18–1.81)	< 0.001
Stage 2/3	36 (2.7)	13 (3.4)	13 (4.7)	0.170	0.73 (0.43–1.24)	0.248
Vascular complications	123 (9.2)	39 (10.1)	22 (8.0)	0.652	0.94 (0.67–1.31)	0.713
Major	39 (2.9)	14 (3.6)	12 (4.3)	0.428	0.74 (0.44–1.24)	0.255
Minor	84 (6.3)	25 (6.5)	10 (3.6)	0.211	1.09 (0.72–1.66)	0.676
New PPM implantation	175/1167 (15.0)	45/340 (13.2)	43/228 (18.9)	0.180	0.96 (0.72–1.27)	0.753

VARC-2 criteria, Kappetein et al. [45]

*PPM* primary pacemaker

* Adjustment of significant basic parameters (age, ejection fraction, previous CABG, coronary artery disease, major neurological deficits, renal disease and frailty) between patients with and without DM

**Table 3:** Procedural characteristics (72 h) and device success (30 days)

	Without diabetes (n = 1337)	Diabetes on OAD (n = 387)	Diabetes on insulin (n = 276)	p-value	Adjusted OR (95% CI)^†^	Adjusted p-value^†^
Device success (n, %)	1196 (89.5)	346 (89.4)	245 (88.8)	0.944	1.10 (0.64–1.91)	0.731
Gradient ≥ 20 mmHg (n, %)	39 (2.9)	14 (3.6)	7 (2.5)	0.228	1.10 (0.64–1.91)	0.731
Moderate or severe aortic insufficiency (n, %)	19 (1.4)	4 (1.0)	3 (1.1)	0.707	0.77 (0.32–1.87)	0.564
Malposition (n, %)	6 (0.4)	0	6 (2.2)	0.079	1.67 (0.52–5.35)	0.391
Second Valve (n, %)	44 (3.3)	14 (3.6)	7 (2.5)	0.607	0.96 (0.56–1.65)	0.881
Procedural mortality 72 h (n, %)	23 (1.7)	8 (2.1)	9 (3.3)	0.685	1.19 (0.62–2.27)	0.598
Early safety (n, %)	1165 (87.1)	326 (84.2)	234 (84.8)	0.258	1.26 (0.96–1.66)	0.096

^†^Adjustment of significant basic parameters (age, ejection fraction, previous CABG, coronary artery disease, major neurological deficits, renal disease and frailty) between patients with and without DM

### Intermediate and long-term survival

**Fig. 1:**
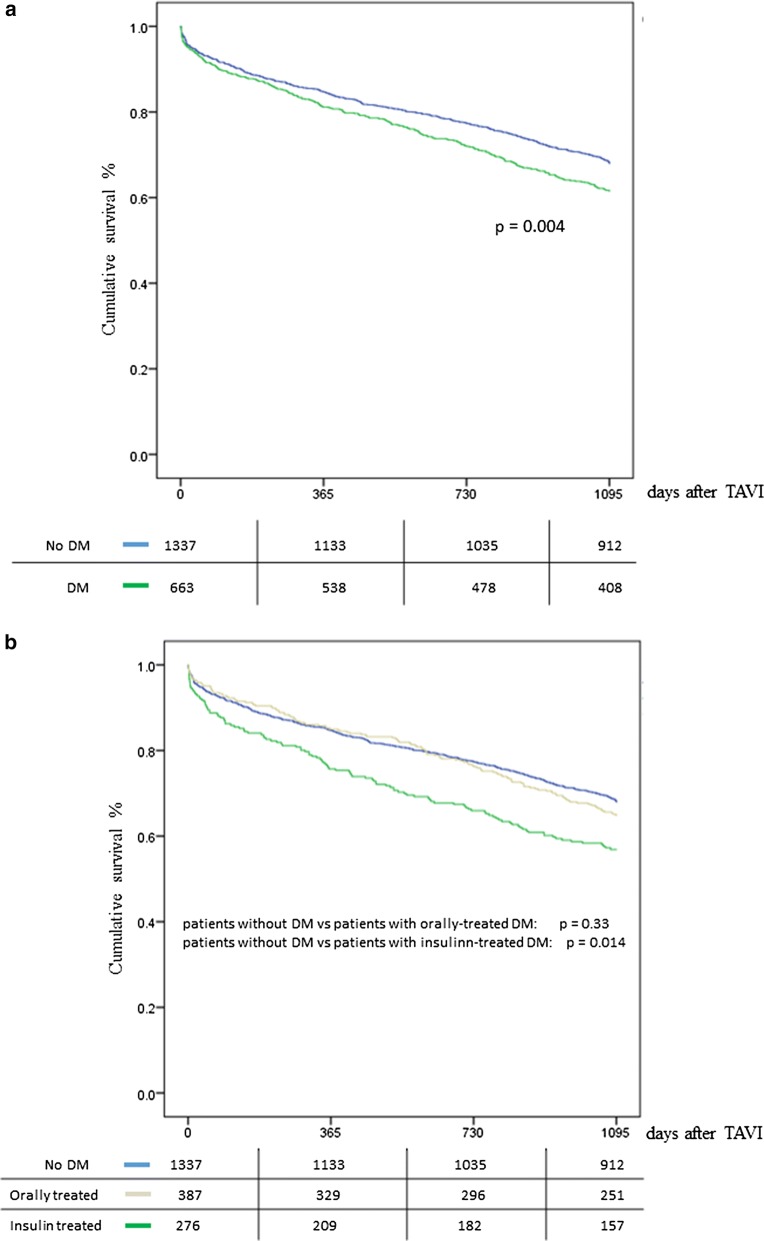
**a** Kaplan–Meier curve; survival rate for patients with and without diabetes. **b** Kaplan–Meier curve; survival rate for patients without patients, with orally-treated and insulin-treated diabetes mellitus. *DM* diabetes mellitus, *OAD* oral antidiabetic drugs

The vital status of the patients was assessed at the end of the 1st year as well as 3 years after the intervention. 1-year survival rate was 84.4% (208 events) for patients without DM and 80.8% (127 events) for patients with DM, suggesting that there was no significant difference between the two groups (p = 0.182). After further division of the patients with DM, the survival of orally treated patients was 84.5% (60 events), while that for insulin-treated patients was 75.7% (67 events), suggesting a statistically significant difference between the last group of patients and both the patients without diabetes (p = 0.003) as well as the orally treated patients (p < 0.001, Fig. 1a, b). 3-year survival rate was 67.9% (429 events) for patients without DM and 61.5% (255 events) for patients with DM, suggesting a significant difference between the two groups (p = 0.004). Following the same analysis pattern, the survival of orally treated patients was 64.9% (136 events) and 56.9% for insulin-treated patients (157 events), suggesting a statistically significant difference between the last patients and both the patients without DM (p < 0.001) as well as the orally treated patients (p = 0.014, Fig. 1a, b).

To further investigate the effect of insulin-treated DM on 1-year survival, we entered this factor in a multivariate Cox regression analysis. Insulin-treated DM was not identified as a statistically significant factor (p = 0.226). The identified significant factors were ejection fraction (p = 0.010), frailty (p = 0.001) and chronic renal failure (p < 0.001), pulmonary hypertension (p = 0.005) and chronic obstructive pulmonary disease (COPD) (p = 0.048) as well as peripheral arterial vascular disease (p = 0.008, Table 4). In year 3, the same factors were identified as statistically significant: ejection fraction (p = 0.001), frailty (p < 0.001), chronic renal failure (p < 0.001), age (p < 0.001) and COPD (p = 0.002), peripheral arterial vascular disease (p = 0.005), pulmonary hypertension (p = 0.001) and previous CABG (p = 0.002).

**Table 4:** Multivariate Cox regression analysis at 1 and 3 years

	1 year		3 years	
	HR (95% CI)	p-value	HR (95% CI)	p-value
Male gender	1.14 (0.90–1.45)	0.274	0.90 (0.76–1.06)	0.217
Age	1.02 (1.002–1.04)	0.031	1.03 (1.01–1.04)	< 0.001
Diabetes				
Orally treated	0.89 (0.67–1.19)	0.452	0.99 (0.82–1.21)	0.935
Insulin treated	1.29 (0.97–1.72)	0.084	1.21 (0.98–1.49)	0.079
EF	0.98 (0.98–0.99)	< 0.001	0.99 (0.99–1.0)	0.001
PAD	1.44 (1.10–1.90)	0.008	1.32 (1.09–1.60)	0.005
Carotid stenosis	1.02 (0.77–1.35)	0.904	0.99 (0.81–1.20)	0.902
COPD	1.37 (1.003–1.87)	0.048	1.41 (1.14–1.74)	0.002
Previous valve surgery	1.13 (0.65–1.94)	0.667	0.97 (0.65–1.46)	0.898
Major neurological deficits	1.08 (0.77–1.51)	0.644	1.13 (0.89–1.43)	0.302
Pulmonary hypertension	1.42 (1.11–1.81)	0.005	1.35 (1.13–1.61)	0.001
Previous MI	1.18 (0.86–1.60)	0.308	1.08 (0.86–1.35)	0.535
Previous CABG	1.13 (0.83–1.55)	0.439	1.40 (1.13–1.73)	0.002
Mitral valve disease	1.26 (0.95–1.68)	0.104	1.07 (0.87–1.33)	0.525
Frailty	1.77 (1.41–2.23)	< 0.001	1.77 (1.51–2.08)	< 0.001
Chronic renal failure	2.65 (1.89–3.73)	< 0.001	2.39 (1.85–3.09)	< 0.001
CAD	0.99 (0.77–1.28)	0.963	1.07 (0.90–1.29)	0.397

*CABG* coronary artery bypass graft, *CAD* coronary artery disease, *LVEF* left ventricular ejection fraction, *MI* myocardial infarction, *PAD* peripheral artery disease, *COPD* chronic obstructive pulmonary disease

## Discussion

Our data suggest that DM is not associated with increased complication risk after TAVI and that insulin-dependent DM was not identified as an independent risk factor associated with increased mortality at 1 and 3 years. In particular, there was no evidence of a significant difference concerning major vascular complications, major stroke or severe kidney injury. Additionally, we had a predominant use of transfemoral access, which is in line with the general preference due to the lesser degree of invasiveness and can generally be managed safely in patients with and without DM [26, 27]. The most common statistically significant complications included minor renal and minor neurological complications. Furthermore, the periprocedural mortality did not differ between patients without diabetes and insulin-treated patients with diabetes, even after adjusting for the different demographic characteristics at baseline. Our results are concordant with other single- and multicenter studies which demonstrate similar survival rates and complications in patients with and without diabetes after TAVI at 30 days after the intervention [15, 28–31]. Similar results were also published for patients with type 2 DM undergoing surgical aortic valve replacement with mechanical and bioprosthetic valve [32]. Those data from a large national registry suggested that in-hospital mortality was not higher for patients with DM.

On the contrary, data from a national multicenter registry (OBSERVANT investigators) identified DM as a significant factor for 30-day mortality [33]. In this study, however, more than 65% of the patients were in New York Heart Association (NYHA) stage III/IV, and there was no stratification between orally- and insulin-treated patients. Furthermore, this discrepancy is probably associated, to a certain degree, with the use of the first-generation devices as well as with the transapical access from the implantation center [23].

### Intermediate and long-term mortality

Concerning mid-term and long-term survival, lower survival rate was observed for patients with diabetes than for those without diabetes; subgroup analysis revealed that this result was mainly driven by insulin-treated patients with DM. This result remained statistically significant even after adjusting for baseline parameters. DM on insulin itself was not identified as a statistically significant parameter for lower survival at 1 year after TAVI in the Cox regression analysis. This result is concordant with the data from a national multicenter registry by Conrotto et al. [18] and a meta-analysis by Ando et al. [34] as well as a multicenter study with data from the society of thoracic surgeons and American college of cardiology transcatheter valve registry published by Abramowitz et al. [35]. The meta-analysis by Ando et al. demonstrated that DM is independently associated with higher midterm mortality but similar perioperative complications, regardless of the type of DM [36], while Conrotto et al. showed that this phenomenon concerns only the insulin-treated group. Finally, Abramowitz et al. indicated a stronger 1-year mortality association in insulin-treated DM than in non-insulin-treated DM.

These findings are also in agreement with those provided from a recent meta-analysis, which identified insulin-treated DM as an independent predictor for poor medium- to long-term outcomes, but was not associated with a higher 30-day mortality [37], while another meta-analysis by Sun et al. [38] suggested that DM was not associated neither with increased risk of periprocedural complications nor with lower survival rate at 30 days and 1 year. In this latest analysis, however, a subgroup analysis for patients treated with insulin was not performed. This finding probably indicates the multifactorial effect of systemic disease, suggesting the need for cardiac risk factor modification for insulin-treated patients with DM, to the extent that this phenomenon could be reflected in the levels of HbA1c [15, 19]. Gu et al. [39] also indicated that HbA1c variability was related independently to the risk of all-cause mortality in patients with heart failure. Finally, the higher incidence of comorbidities, such as coronary artery disease and impaired renal function, could possibly contribute to the increased mortality risk in patients treated with insulin. Another study suggested that the gender itself might also play a role to the clinical outcomes [40]. The pathophysiological background of these clinical findings might include the procoagulant imbalance, the chronic exposure to high glucose levels and the effects of hyperinsulinemia, which are reflected, to a certain degree, to the use of insulin. The role of coronary artery disease is also well-established in the current literature, suggesting that DM and especially insulin therapy, is a strong predictor for cardiovascular mortality as well as particularly for late or repeat revascularization irrespective of an early procedure [41]. The same mechanism may also be partially an explanation for increased mortality in patients with DM after major cardiovascular events, such as stroke, aortic aneurysm and dissection, acute lower limb ischemia and myocardial infraction, as indicated from a large national registry [42].

### The use of risk scores in TAVI

Diabetes mellitus is integrated in the STS risk score, and insulin-treated DM is integrated in the EuroSCORE II [43, 44], with satisfactory performance predicting cardiac surgical perioperative and 30-day mortality; however, these two scores do not indicate a relevant discrimination for the procedural outcome. The logistic EuroSCORE, on the other hand, does not include diabetes as a factor. Several studies have indicated that 30-day mortality may be more accurately predicted by the EuroSCORE II than by the other two risk scores, suggesting that this risk score is a valuable tool in the clinical setting [43, 44]. The update of the EuroSCORE derived from the need for better calibration, as risk-adjusted cardiac surgical mortality has declined in recent decades. The researchers of the working group provided evidence that only insulin-dependent DM was associated with higher mortality. A EuroSCORE II validation study by Barili et al. also suggested that diabetes on insulin is a significant risk factor for open surgical perioperative risk, while other factors, such as NYHA II, pulmonary and neuromuscular dysfunction as well as pulmonary artery systolic pressure < 55 mmHg, could be removed from the risk model without changing the performance of the model [21].

The discrepancy in the role of diabetes in the assessment of perioperative risk in TAVI could be explained by the inherited characteristics of the risk assessment tool. The current perioperative risk scores derive from open surgical registries and are designed mostly to predict the risk of surgical aortic valve implantation but not TAVI. Supposedly, a model risk score specifically designed for TAVI could weigh discriminative factors other than those of the current scores. The Placement of AoRTic TraNscathetER Valve (PARTNER) trial indeed showed that intermediate risk patients undergoing transfemoral TAVI had better outcomes than those of open surgical patients [8], and the extended use of local anesthesia has improved the procedural outcomes, which consequently indicates a risk stratification for access in TAVI. Therefore, the use of the current perioperative risk tools not only is useful for risk estimation in TAVI but also implicates the prominent need for the development of more comprehensive TAVI risk tools.

### Limitations

The main research limitation of this study is the observational nature and the resulting potential for variability. The standardized protocols used at our TAVI center are likely to have minimized disparities; nevertheless, the role of unmeasured confounders cannot be ruled out. Good-quality readily available data improve the validity of comparisons. Furthermore, this is a single center analysis and, therefore, there might be an unavoidable risk for bias regarding treatment options. However, the sample size of this registry might improve the external validity of the results. Finally, it was possible to distinguish the all-cause mortality into cardiovascular and non-cardiovascular mortality in a reliable way, particularly because of the large size of the registry and the wide time span in which the registry was conducted. Further information, such as the glycemic control of the disease, was not collected, implying a possible effect of the control itself rather than the choice of insulin use.

## Conclusions

Although insulin-dependent DM is an established component of surgical risk assessment, it was not identified as an independent factor associated with reduced survival in TAVI. DM treated with OAD or insulin may have less role in decision making of treatment in TAVI candidates.

## Data Availability

The datasets generated and/or analyzed during the current study are not publicly available due to the European data protection regulation but are available from the corresponding author on reasonable request.

## Abbreviations

adjOR: adjusted odds ratio
AKI: acute kidney injury
CI: confidence interval
CABG: coronary artery bypass graft
CAD: coronary artery disease
COPD: chronic obstructive pulmonary disease
DM: diabetes mellitus
LVEF: left ventricular ejection fraction
MI: myocardial infarction
NYHA: New York Heart Association
OAD: oral antidiabetic drugs
OR: odds ratio
PAD: peripheral arterial disease
PPM: primary pacemaker
SAVR: surgical aortic valve replacement
SD: standard deviation
STS: Society of thoracic surgery
TAVI: transcatheter aortic valve implantation
TAVIK: TAVI team Karlsruhe
VARC: valve academic research consortium
